# Asking questions can help: development and preliminary evaluation of a question prompt list for palliative care patients

**DOI:** 10.1038/sj.bjc.6601380

**Published:** 2003-11-25

**Authors:** J Clayton, P Butow, M Tattersall, R Chye, M Noel, J M Davis, P Glare

**Affiliations:** 1 Medical Psychology Research Unit, Blackburn Building D06, University of Sydney, NSW 2006, Australia; 2 Sacred Heart Palliative Care Service, St Vincent's Hospital, Darlinghurst, Sydney, NSW 2010, Australia; 3 Department of Cancer Medicine, Blackburn Building D06, University of Sydney, NSW 2006, Australia; 4 Palliative Care Service, Nepean Hospital, Penrith, NSW 2751; 5 Palliative Care Service, Calvary Hospital, Kogarah, Sydney, NSW 1485, Australia; 6 Department of Palliative Care, Royal Prince Alfred Hospital, Camperdown, Sydney, NSW 2050, Australia

**Keywords:** palliative care, communication, patient participation, physician patient relations, truth disclosure, question prompt list

## Abstract

Question prompt lists (QPLs) have been shown to be an inexpensive and effective communication tool for patients in oncology consultations. We aimed to develop and pilot a QPL for palliative care (PC) patients. In order to identify suitable questions for inclusion in the QPL, we conducted focus groups and individual interviews with 19 patients, 24 carers and 22 PC health professionals. A further 21 health professionals reviewed the draft document. The draft QPL was piloted in 23 patients. In total, 112 questions were identified and grouped into eight categories. All participants felt that the QPL, in booklet form, could be a useful tool. Out of 23 patients in the pilot study, 22 agreed that the QPL was helpful, contained useful questions, was easy to understand and would be useful in the future. State anxiety (STAI) decreased after receiving the booklet and seeing the doctor in 16 out of 19 patients (overall anxiety decreased by a median of 8, IQR 1–13). Participants in the pilot study endorsed the inclusion of end-of-life issues in the QPL, despite some reservations expressed about this by health professionals in the individual interviews. We have identified a specific QPL that might facilitate useful dialogue between PC patients and their doctor. The QPL has strong support from patients, their carers and relevant health professionals.

Optimal communication has been identified by patients and their families as one of the most important aspects of medical care at the end-of-life (Steinhauser *et al*, 2000; Curtis *et al*, 2001; Wenrich *et al*, 2001). Medical practitioners tend to underestimate the information needs of cancer patients (Degner *et al*, 1997a). The information needs of individual patients vary (Leydon *et al*, 2000; Jenkins *et al*, 2001). Hence, a blanket policy of fully informing and involving all patients may not best serve their interests. Communication may be improved when patients are able to ask questions that are of greatest concern to them. Some health professionals encourage patients to write down their questions and bring them to medical appointments, but patients may not know what questions to ask or how to articulate their concerns. Butow *et al* (1994) explored the use of a question prompt list (QPL) given to cancer patients before their initial consultation with oncologists. A QPL is a structured list of questions for the patient to ask the doctor if they wish. It is designed to encourage patient participation during a medical consultation and to assist patients in acquiring information that is suited to their needs and at their own pace. This simple and inexpensive intervention has been found to promote question asking about prognosis in three separate studies (Butow *et al*, 1994; Brown *et al*, 1999, 2001). In the most recent of these studies (Brown *et al*, 2001), provided the oncologist specifically addressed questions in the QPL during the consultation, those patients who received the prompt list were significantly less anxious immediately after the consultation and had better recall and significantly shorter consultations.

Patients in a palliative care (PC) setting may also benefit from receiving a QPL. The specific information needs of patients being referred to a specialist PC service are not well documented. The current study aimed to determine the information needs of PC patients in order to develop an evidence-based QPL specific to this setting and to pilot this QPL in patients attending a specialist PC service.

## MATERIALS AND METHODS

### Participants

Three groups felt to have important input were sampled: (a) PC patients, (b) carers of PC patients, and (c) health professionals working in PC. Patients and carers were eligible to take part if they were: (1) over 18 years of age, (2) English speaking, (3) well enough to participate, (4) able to give informed consent, and (5) referred to a specialist PC service and diagnosed with an incurable and progressive illness, or the carer of such a patient. In all, 19 patients and 24 carers were recruited from hospitals, PC units and the community in three PC services in Sydney. Their demographic characteristics are shown in Table 1

**Table 1:** Demographic and disease characteristics of patient and carer participants

**Characteristic**	**Carers participating in focus group/individual interviews (*n*=24)**	**Patients participating in focus group/individual interviews (*n*=19)**	**Patients participating in pilot study (*n*=23)**
*Age (years)*			
<60	17	7	8
>60	7	12	15
			
*Sex*			
Male	8	8	11
Female	16	11	12
			
*Education*			
School certificate or below	9	3	18
Completed high school but not tertiary	1	5	2
Tertiary education	14	11	3
			
*Underlying diagnosis of patient*			
Advanced malignancy	23	19	23
Other	1	0	0
			
*Doctor's estimate of patients' survival*			
<4 weeks			1
4–12 weeks			7
>12 weeks			15
			
*Previous contact with PC doctor*			
Nil prior contact			14
Seen one time before			6
Seen two times before			3
			
*Previous contact with PC nurse*			
Nil contact so far			11
Seen 1 time before			3
Seen 2 times before			4
Seen >2 times before			5
			
*Reason for consultation*			
Initial link up with PC			5
Physical symptoms or psychosocial issues			14
Routine follow-up			4
			
*Preferences for decisional control*			
Passive			8
Collaborative			13
Active			2
			
*Preferences for information*			
Want information needed to care for myself properly			2
Want additional information only if good news			2
Want as much information as possible, good and bad			19

.

In total, 22 PC health professionals were interviewed, including seven senior doctors, six senior registrars in training, four nurses and five allied health staff. They worked at 10 PC services in two states of Australia in teaching hospital, community and inpatient PC unit settings. Seven (32%) had more than 10 and all more than 2 years experience. A further 21 health professionals, including PC doctors and nurses, Family Medical Practitioners, a medical oncologist and an expert in consumer materials reviewed the draft QPL. We piloted the final draft QPL in 23 patients seeing one of three PC doctors from three services in Sydney (see Table 1).

### Data collection and analysis

Focus groups of four to eight participants, supplemented by individual interviews with those unable to attend a focus group, were held separately with patients and carers and conducted by a PC physician (JC) and a clinical psychologist (PB). Health professionals were given a semistructured individual interview either face to face or over the telephone. For patients and carers, the discussion explored the meaning of PC, questions they had asked the PC service and questions they wished they had asked. The interview for health professionals explored the information thought to be most important to convey to patients during PC consultations, common questions asked by patients and their carers, and questions they felt would elicit useful information, but patients and carers may have difficulty asking. All participants were asked their views on the QPL and when it should be given.

The focus groups and telephone interviews were audiotaped and fully transcribed. Data analysis was informed by qualitative methodology (Pope and Mays, 2000). Individual questions or information needs were identified from the transcripts using the participants' own language where possible. These were discussed by both facilitators to ensure consistency of interpretation and were organised into categories to facilitate comprehension, as suggested by Ley (1988). Further focus groups and/or telephone interviews were conducted until no additional topics were raised. A draft QPL was developed using the categories and questions identified in the transcripts. The draft QPL was then reviewed by relevant health professionals and their suggestions were incorporated. The document was further refined to ensure ease of understanding and a Flesch–Kincaid reading grade level score of below 8.0.

Based on feedback regarding optimal timing of QPL provision derived from focus group and interview data, enrolment in the pilot study was within three consultations from initial contact with the PC clinician. The consultations all took place in an outpatient PC clinic.

Prior to the consultation, patients completed a questionnaire regarding their information and involvement (decisional control) preferences, and anxiety levels. General preferences for information were assessed using a question from the Cassileth Information Styles Questionnaire measuring the type of information preferred (Cassileth *et al*, 1980). Preferences for decisional control were assessed using a validated and reliable question from previous studies in cancer patients (Sutherland *et al*, 1989; Degner *et al*, 1997b). Patients were classified as wanting an ‘active’, ‘passive’ or ‘collaborative’ role when making decisions about treatment. Patient anxiety was measured by the Spielberger State Anxiety Inventory (Spielberger, 1983) (20 items), which produces a continuous score (range 20–80) with higher values representing higher anxiety levels. The latter is a widely used scale measuring situational anxiety.

Patients had about 20 min to review the QPL. Clinicians were asked to endorse and refer to the QPL during the consultation.

After each consultation, clinicians were asked whether the QPL interfered with the flow of the consultation (Yes/No/Not sure) and whether they thought that it made it any easier for either the patient or themselves to raise certain issues during the consultation (Yes/No/Not sure). If clinicians answered yes to the latter they were asked to comment. After the consultation, patients also completed a questionnaire measuring anxiety and responses to the QPL using standard items.

After 3 weeks, patients completed a further questionnaire regarding the usefulness of the QPL during subsequent contacts with the PC team where that had occurred. At the completion of the study, participating clinicians completed a questionnaire eliciting attitudes towards the QPL, and whether they would incorporate it into their own practice.

The study was approved by the ethics committees of participating institutions.

## RESULTS

### Results of focus groups and individual interviews

Eight categories of questions were identified from the transcripts.

#### About the PC service and team

General questions included how and when to contact the PC team, and the connection between the PC team and other health professionals involved in the patients' care. In addition, some patients wanted advice about how to choose another doctor. A second opinion was valued regarding disease-specific treatment from an independent source. Others wanted to discuss the option of stopping anticancer treatment or not starting it at all.

#### Physical symptoms and treatment

**Figure 1:**
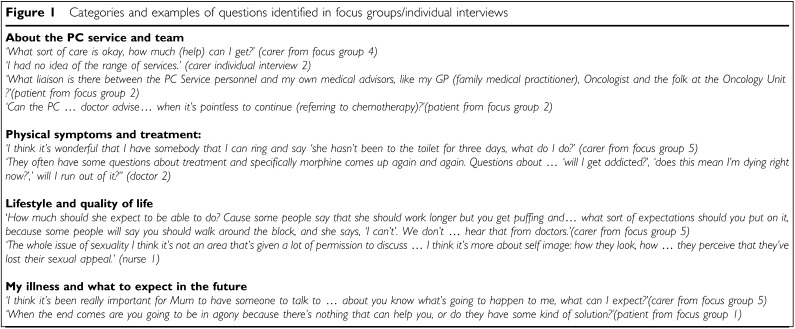
Categories and examples of questions identified in focus groups/individual interviews

Most participants suggested questions about the management of a range of physical symptoms (see Figure 1

). Several very detailed questions were raised about medications, particularly morphine.

#### Lifestyle and quality of life

Participants wanted advice about how to make the most of their life (see Figure 1). Several were not sure how physically active they could expect to be. Intimacy and body image were raised by several health professionals as topics patients have difficulty asking about, but are sometimes relieved to discuss. These latter topics were not raised by patients or carers.

#### My illness and what to expect in the future

Several questions were raised about underlying disease and prognosis (see Figure 1). Pain was a particular concern in the future and around the time of death.

#### Support

**Figure 2:**
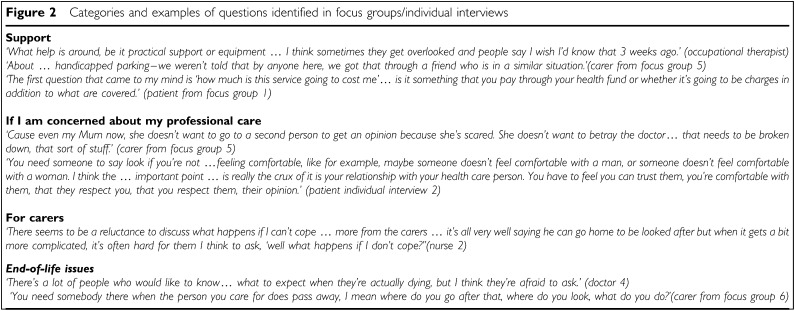
Categories and examples of questions identified in focus groups/individual interviews

Many participants raised the requirement for support in various forms for both carers and the patient themselves (see Figure 2

). All participant groups commented that simple practical issues could be overlooked in early consultations with the PC team, particularly if there were major physical symptoms or emotional concerns to address. Several patients and carers stated that they found out about disability parking some time after struggling to get to appointments. The cost of care was also raised. Patients and carers admitted to feeling uncomfortable asking such questions for fear of ‘quibbling’ over costs. Some health professionals, but no patients or carers, suggested questions about spiritual and cultural issues.

#### If I am concerned about my professional care

Patients and carers were uncertain what to do if they had a concern about their care or an aspect of the PC service (see Figure 2). This issue was not raised by any health professionals.

#### For carers

Carers and health professionals raised questions related to the role of the carer (see Figure 2). Carers were anxious about what skills would be required to take care of the person at home. Both carers and health professionals stated that carers may be hesitant to voice concerns about their ability to cope.

#### End-of-life issues

Questions about end-of-life issues were raised mainly by carers and health professionals (see Figure 2). While many suggested that these would be important to include, concern was expressed by some health professionals that these issues could be confronting for patients, especially at the time of initial referral to PC.

#### Additional comments and recommendations for a QPL

**Figure 3:**
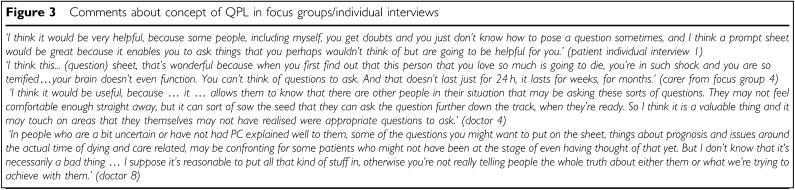
Comments about concept of QPL in focus groups/individual interviews

All patients and carers felt that the QPL would be useful (see Figure 3

). Of 22 health professionals, 20 were very positive about the concept of the QPL (see Figure 3). Two health professionals thought that it could be useful but confronting, especially for patients referred early in the course of their illness, who may not be ready to think about noncurative treatment.

Participants recommended a written introduction to the QPL defining PC, endorsing question asking during the current and subsequent consultations, encouraging people to keep the QPL for future reference and reassuring patients that not all questions may be relevant to them. Most patients and carers and approximately half of the health professionals felt that the prompt list should be given prior to or during the first consultation with the PC team. The remainder felt that the QPL should be given later, for example, at the second or third consultation.

A total of 104 questions within eight categories were identified via the focus groups and individual interviews. Eight additional questions were added as a result of feedback from health professionals. The final draft QPL was a booklet titled ‘Asking questions can help: an aid for people seeing the PC team’ (see Appendix A).

### Results of pilot study

Of the 23 participants, 20 completed their post consultation questionnaires, the remaining three became acutely unwell but gave verbal feedback to the research nurse following the consultation. In all, 11 patients completed the 3-week follow-up questionnaires (five patients were too unwell, four died, two did not respond to a reminder and one patient's carer requested no further involvement).

#### Patient feedback on QPL from pilot study

Patients' views about the QPL are outlined in Table 2

**Table 2:** Patient and carer feedback about QPL from pilot study

		**Post consultation questionnaire refers to consultation immediately after receiving QPL (*n*=20)**			**3-week follow-up refers to use of QPL for subsequent contacts with PC team (*n*=11)**		
		I disagree or disagree completely	I don't know	I agree or agree completely	I disagree or disagree completely	I don't know	I agree or agree completely
I found brochure to be helpful		1		19			11
The brochure made it easier to ask questions		1	2	17		1	10
There were questions in brochure that were useful to me		1		19			11
There were questions in brochure that made me anxious		10	2	8	7	3	1
The brochure helped me to put some of my questions or concerns into words		1	3	16	1	1	9
I found it overwhelming to read the brochure		13	2	5	10	1	
I think the brochure will be useful to me in future			1	19		2	9
The brochure was easy to understand		1		19			
						
Views on length of QPL	Right length		16				
	Too long		3				
	Too short		1				
							
Did you have enough time to read the booklet before consultation?	Yes		16				
	No		3				
	Unsure		1				
							
Would you have preferred to receive the booklet at different time?	Yes		9^a^				
	No		11				
							
Have you read the booklet again since first receiving it?	Several times					2	
	1–2 times					6	
	Not at all					3	
							
Did the booklet prompt you to ask your PC doctor any questions?	Yes		17			6	
	No		1			0	
	Unsure		1			0	
	Other		1^b^			1^b^, 4^c^	
							
Did the booklet prompt you to ask questions of other members of PC team?	Yes					5^d^	
	No					4	
	Other					2^c^	
							
Did anyone else read the booklet (i.e. carer/relative or friend)?	Yes		16			11	
	No		4			0	
							
If anyone else read the booklet was it helpful to them?	Very helpful		10			4	
	Bit helpful		6			5	
	Not helpful		0			0	
	Not sure		0			2	

a Seven patients would have preferred if QPL was mailed to them 2–3 days before consultation, two patients would have preferred to have received QPL earlier in the course of their illness.

b Asked some of the questions, but felt they would have asked them anyway without having read the booklet.

c Had not seen the doctor/and or palliative care team member since the day of initial consultation.

d Discussed questions with a nurse in three cases, in two cases the patient did not indicate which other PC heath professional they discussed the questions with.

and Figure 4

**Figure 4:**
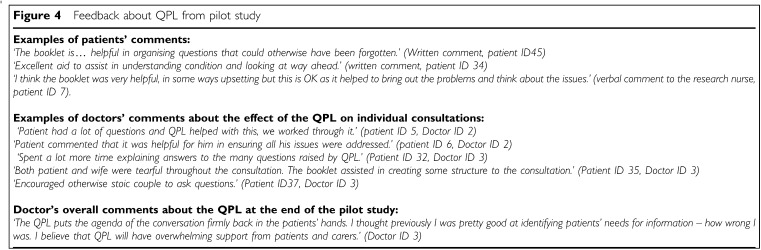
Feedback about QPL from pilot study

. Only one patient responded negatively, more to the process of participating in research than to the QPL itself.

Anxiety scores decreased after receiving the booklet and seeing the doctor in 16 patients and increased by a minimal amount in two patients and more substantially in one (increase of 20 points). The overall anxiety decreased by a median of 8 (IQR=1–13).

Patients suggested removing only one question: ‘How can you make sure that I have died?’, but endorsed a similar question for carers ‘How do I know when he/she has died?’ and the other end-of-life questions, even if they themselves did not want to ask such questions in the current consultation. Some patients specifically said that they valued the end-of-life section and that it made it easier to raise these issues with their doctor. The carer of one patient who died 1 week after participating in the study contacted us to say that the QPL acted as a catalyst for discussion about end-of-life issues that they had not been able to broach previously. No additional questions were suggested.

#### Doctor feedback on QPL from pilot study

In all 23 cases, the doctor indicated the QPL did not interfere with the flow of the consultation. In 20 cases, the doctor felt that the QPL made it easier for either the patient or themselves to raise certain issues during the consultation such as end-of-life issues or prognosis. Other discussion topics that doctors felt were prompted by the QPL included diet, financial problems, the relationship between the PC team and their general practitioner and the availability of support services. In the questionnaire after the completion of patient recruitment, all three participating doctors said that the QPL was a very useful tool for patients, none had reservations about the QPL and all felt that they would use the QPL for patients in the future (see Figure 4).

## DISCUSSION

This study aimed to identify a list of questions to assist patients' understanding of PC and to facilitate achievement of their information needs and involvement in decisions about their care. Analysis of these data identified 112 questions within eight categories, see Appendix A.

The information and supportive care issues raised during the focus groups and interviews are similar to those identified in previous research of cancer patients' unmet needs (Sanson-Fisher *et al*, 2000). However, our results identified many needs particular to the PC setting; for example, questions about the amount of help available for carers and about medications, particularly morphine. Secondly, issues not previously discussed in the literature were raised, such as patients' and carers' need for reassurance that they can get a second opinion if they wish, either to hear another view or because they are uncomfortable in some way with their current doctor or nurse. Finally, carers and health professionals raised end-of-life issues as a sensitive area that patients may be frightened to ask about, but relieved to be able to discuss.

There were some issues raised by patients and carers, but not by health professionals, suggesting that health professionals are not aware of patients' need for information in these areas. For example, what patients should do if they are concerned about an aspect of their PC and whether they can seek a second PC opinion, about handicap parking, very detailed questions about medications and the costs of care. Likewise, there were some issues that health professionals raised that were not specifically brought up by patients or carers, namely sexuality, body image and spiritual issues. The reason why patients and carers did not raise these issues is not clear. Perhaps, they were not comfortable discussing this in a focus group setting or even in a confidential individual interview. Possibly they are not topics they would think of discussing with their doctor or nurse. In the case of sexuality, the patients may have been so unwell that they had other priorities.

All patients and most health professionals taking part in the focus groups and individual interviews felt the question list, in booklet form, could be a useful tool for patients seeing the PC team. In the pilot study, we evaluated the content validity, acceptability and perceived usefulness of the QPL for PC patients. All doctors and 22 of 23 participating patients were very positive about the QPL in making it easier to ask questions and discuss issues of concern. Overall, there was a reduction in anxiety scores. Whether this was a result of seeing the doctor or the use of the QPL is unclear from the design of the study, but it does suggest that the process of reading the booklet and seeing the doctor did not unduly increase anxiety. The possibility of this intervention raising anxiety had been a concern and this result is reassuring. Despite health professionals' previously expressed concern about the inclusion of questions about end-of-life issues in the booklet, these questions were endorsed in the pilot study and were felt to be of particular benefit by both patients and participating doctors.

### Limitations and applicability of findings

In the pilot study, the majority (82.6%) of participating patients stated prior to the consultation that they wanted all possible information, be it good or bad news. This is similar to the information preferences reported in cancer patients receiving treatment with palliative intent (Fallowfield *et al*, 2002). The educational background of patients participating in the pilot study was generally lower than those taking part in the focus groups and individual interviews. Yet, all but one patient in the pilot study reported that the booklet was easy to understand. Patients from three different PC services in Sydney took part in the study. However, all patients in the pilot study were English speaking and were being reviewed in an outpatient-clinic setting. A significant proportion of patients referred to PC in Sydney and Australia come from non-English-speaking backgrounds and are reviewed by the PC doctor either in their own home or in the hospital.

### Implications and future research

We have identified a specific set of questions that patients, carers and health professionals felt might facilitate useful dialogue between patients and their PC doctor. This question list, in the form of a booklet, appears to be a beneficial tool for patients and carers both in the early interactions with the PC team and as a future reference. The concept has strong support from relevant health professionals. However, the study is limited to three PC services in Sydney. Before widespread adoption of the intervention a larger scale evaluation, with the inclusion of a control group, would be appropriate. The modification of the prompt list for different cultural groups and its use by other members of the PC team also merits investigation.

## Appendix A

### ASKING QUESTIONS CAN HELP: AN AID FOR PEOPLE SEEING THE PALLATIVE CARE TEAM

#### **About the pallative care service and team**

##### Available care

Who are the members of the pallative care team and what do they do?
What does the pallative care service offer that is different from the services provided by the other doctors/nurses that I see?
Can I see the pallative care team both when I am at home and when I am in the hospital?
How much help is available at home (e.g. how often can I be seen by the pallative care team)?
What do pallative care hospitals offer?
Is it possible for me to be admitted to the pallative care hospital for a short time (e.g. to get my symptoms under control or to give my family a break), and to then go home again?
How do I access the services offered by the pallative care team?
What is the cost involved with seeing the pallative care team?

##### Contacting the pallative care team

How can I contact the pallative care team?
In what circumstances can I or should I contact them?
How often can I contact them?
Is the pallative care service available after hours or in emergencies?

##### Relationship between the pallative care team and other health professionals

Does the pallative care team speak to or write to my GP and other specialists about my care?
What is the role of my GP now that I have been referred to the pallative care team?
Which of my health professionals should I contact first if I am unwell or if there is an emergency?
Who will see me on a regular basis from now on?
Can you help me choose a GP or another specialist?
Can you help me work out questions that I may wish to ask my other doctors/specialists?
Can you give me advice about treatment decisions that I am discussing with other doctors? For example, whether to stop or start chemotherapy or other treatments.

#### **Physical symptoms**

If I have symptoms, what can be done to improve them? (e.g. pain or discomfort, constipation, shortness of breath, nausea or feeling sick, lack of appetite, tiredness, dry mouth)
Can you help control my pain?
What are the different options available for controlling my pain?
Can you help control my other symptoms?
What is the cause of my symptoms?

#### **Treatment**

##### Medications

Please tell me the side effects of any new medication you prescribe. How likely are they to occur?
What can be done about these side effects?
Will new medication affect any of my present medications or other medical conditions?
Are there any tablets that I should NOT take while on this new medication?
What is the cost of any new medication?
Can I get the new medication from my local pharmacy?
What are all my tablets for?
Are all my old tablets still necessary?
How and when should I take my medication?
How can I manage to take all my medication?
Are there any natural or complementary (alternative) therapies that may be helpful for me?

##### Morphine

Will my body get used to morphine if I start it now?
Will it still be effective in the future?
Is it addictive?
Can I stop taking it if my pain goes away?
Will it make me confused or sleepy?
Will it make me constipated?
What are the different ways of taking morphine (e.g. tablets)?
Are there other painkillers or alternatives available?

#### **Lifestyle and quality of life**

What can I expect to be able to do?
How much activity or exercise is too much and how much is too little?
What activities may help me enjoy life more, for example, massage, meditation?
How can I make the most of my life?
What kind of food should I eat?
How important is my diet?
Can you advise me if and when I can return to work?
Can you advise me about the timing of a holiday or trip I wish to take?
Is it OK for me to drive?
How can I remain close and intimate with my partner (physically and/or emotionally)?

#### **My illness and what to expect in the future**

What is going on with my illness?
What are the chances of controlling my illness?
Will the illness progress?
What can I expect in the future?
What symptoms may occur in the future and what should I do if they arise?
Will I be in pain?
Will you be able to control my pain and other symptoms in the future?
What are the worst days going to be like?
What are the best days going to be like?
How long am I likely to live?

#### **Support**

##### Support in the form of information

What information is available about pallative care and my illness?
Are books, videos or pamphlets available?
Are there any other organisations that would be useful for me to contact?

##### Practical support

Is there a programme of activities available through the pallative care service? (e.g. physiotherapy, massage, spa, breathlessness clinic, day centre)
Can you provide equipment to make everyday living easier at home?
Am I eligible for disability parking? How do I apply for this?
Are there any volunteers available to help me? (e.g. to take me to an appointment or to do the shopping)

##### Financial support

What costs will I have during my illness (e.g. for any equipment required or medications)?
Is there any way I can get medical equipment (e.g. oxygen) or medications at a cheaper price?
What financial assistance is available for my carer or me (e.g. pensions)?
Is there someone I can talk to about financial matters?

##### Emotional support

How am I likely to feel through this and what can I do to cope?
How can I deal with depression if this occurs?
Is there someone I can talk to about my fears and concerns?
How can I cope with the changes in my body as a result of this illness?
Is it possible for me to talk to a member of the pallative care team alone or for my carer to do so?
Are there any support groups available?
Can someone help me communicate with other members of my family about what is happening to me?
What support is available for other people in the family, such as my carer or my children?

##### Spiritual and cultural support

Is there anyone that I can speak to about my spiritual or religious needs?
Can you arrange for me to talk with someone from my culture, someone who may understand me better?

### IF I AM CONCERNED ABOUT MY PROFESSIONAL CARE

Who can I talk to if I am concerned about the care that I am receiving?
Is it possible for me to see someone else if I don't g*et al*ong with my pallative care nurse or doctor? How do I go about this?
Can I get a second opinion about any aspect of my pallative care?
Can I choose which hospital or pallative care team I am linked to?

#### **For carers**

What skills will I need as a carer?
Do you think I can look after my partner, relative or friend at home?
Can I get help if I cannot manage?
What can I do if I am not coping?
How can I best support the person that I am caring for?
What should I do if my partner, relative or friend won't eat very much?
If my partner, relative or friend eats more, will this make them live longer?
How can I assist health professionals to talk to my partner, relative or friend in a way that respects their personality/culture?
Who can I talk to if I am concerned about the care my partner, relative or friend is receiving?

#### **End of life issues**

##### Questions that I may like to ask

How do I get my affairs in order and write a will?
Who can I talk to about the medical care that I want in the future when I am no longer able to speak for myself?
How can I cope when I get sicker and can no longer care for myself?
How can I cope with becoming more dependent on others?
What can I expect in the last days of my life?
Will you be able to tell me when it is getting close to the time I will die?
What happens if I go into a coma?
Is it feasible for me to die at home rather than in the pallative care ward or hospital?

##### Questions that my carer or family may like to ask

If I cannot manage to look after my partner, relative or friend at home, how can we come to terms with this?
What should I say when the person that I am caring for asks, ‘am I dying’?
Will you be able to tell me when it is getting close to the time that he/she will die?
When should I call the rest of the family? What should I say to them? Could you speak with them?
How do I know when he/she has died?
What happens after he/she dies (e.g. what happens to their body, how do we arrange the funeral)?
What support is available for the family after the person dies?

#### **Other features of printed booklet**

- Contents page
- Written introduction defining pallative care, endorsing question asking, explaining the purpose of the booklet, suggesting that people may like to circle the questions they want to ask their doctor or nurse, suggesting that the booklet is kept for future reference and that patients may wish to use it with other members of the pallative care team, explaining that there may be some questions or topics that are not relevant to the patient or their stage of illness and suggesting that patients read the topic headings first and decide whether they want to read questions on that particular topic
- Additional brief introductions to the carer and end of life sections explaining that these topics may not be relevant to the patient or their stage of illness.
- Spaces throughout booklet for patients to write additional questions
- Only one topic printed on each page of booklet
